# Metagenomic surveillance for bacterial tick-borne pathogens using nanopore adaptive sampling

**DOI:** 10.1038/s41598-023-37134-9

**Published:** 2023-07-07

**Authors:** Evan J. Kipp, Laramie L. Lindsey, Benedict Khoo, Christopher Faulk, Jonathan D. Oliver, Peter A. Larsen

**Affiliations:** 1 Department of Veterinary and Biomedical Sciences, College of Veterinary Medicine, University of Minnesota—Twin Cities, St. Paul, MN USA; 2 Division of Environmental Health Sciences, School of Public Health, University of Minnesota—Twin Cities, Minneapolis, MN USA; 3 Department of Animal Science, College of Food, Agricultural and Natural Resource Sciences, University of Minnesota—Twin Cities, St. Paul, MN USA

**Keywords:** Pathogens, Next-generation sequencing

## Abstract

Technological and computational advancements in the fields of genomics and bioinformatics are providing exciting new opportunities for pathogen discovery and genomic surveillance. In particular, single-molecule nucleotide sequence data originating from Oxford Nanopore Technologies (ONT) sequencing platforms can be bioinformatically leveraged, in real-time, for enhanced biosurveillance of a vast array of zoonoses. The recently released nanopore adaptive sampling (NAS) strategy facilitates immediate mapping of individual nucleotide molecules to a given reference as each molecule is being sequenced. User-defined thresholds then allow for the retention or rejection of specific molecules, informed by the real-time reference mapping results, as they are physically passing through a given sequencing nanopore. Here, we show how NAS can be used to selectively sequence DNA of multiple bacterial tick-borne pathogens circulating in wild populations of the blacklegged tick vector, *Ixodes scapularis*.

## Introduction

Nanopore sequencing, based on platforms pioneered by Oxford Nanopore Technologies, Inc. (ONT), has proven to be an increasingly valuable tool for a variety of long-read and metagenomic sequencing applications^1^. In brief, nanopore sequencing is accomplished by passing individual DNA (or RNA) molecules through a membrane-embedded protein nanopore; as a strand of nucleic acid moves through the nanopore, changes in ionic current across the membrane are measured and processed into nucleotide sequence data^2–5^. Importantly, the single-molecule nature of nanopore sequencing permits notable advantages over other next-generation sequencing technologies. In particular the palm-sized ONT MinION sequencing instruments are user-friendly, generate real-time results, and are highly portable, thus enabling their use across a wide variety of lab- and field-based settings^6–12^. Furthermore, single-molecule nanopore sequencing can yield long-read sequencing data, with individual sequences measuring many thousands of bases in length, without a requirement for clonal library amplification. This aspect of the nanopore sequencing approach helps to limit potential sequencing biases related to complex or repetitive genomic regions where short-read sequencing-by-synthesis methods (e.g., Illumina) typically struggle^13–15^. Although the per-base accuracy of ONT sequencing remains marginally less than that of other next-generation platforms, steady advances in sequencing chemistry and basecalling have yielded continued and substantial improvements in reliability and overall utility^4,5,16^.

Due in part to these key advantages—ease of library preparation, the small and portable footprint of ONT sequencing instruments, and the generation of real-time sequencing data—nanopore sequencing is particularly well-suited for the purposes of pathogen detection and biosurveillance^15,17–19^. In contrast to conventional surveillance methods, which rely heavily on nucleic acid amplification (i.e., PCR), pathogen detection by nanopore sequencing can: (1) bypass the requirement that investigators possess a priori knowledge of the pathogen(s) being targeted, (2) enable detection of many agents simultaneously, and (3) provide a greater scope of downstream genomic sequence information suitable for pathogen strain typing, functional characterization, and phylogenetics^17,20^. For these reasons, metagenomic and metatranscriptomic approaches using nanopore sequencing offer an innovative and unbiased means for detecting and characterizing a wide variety of pathogens relevant to both human and animal health^20–22^.

**Figure 1:**
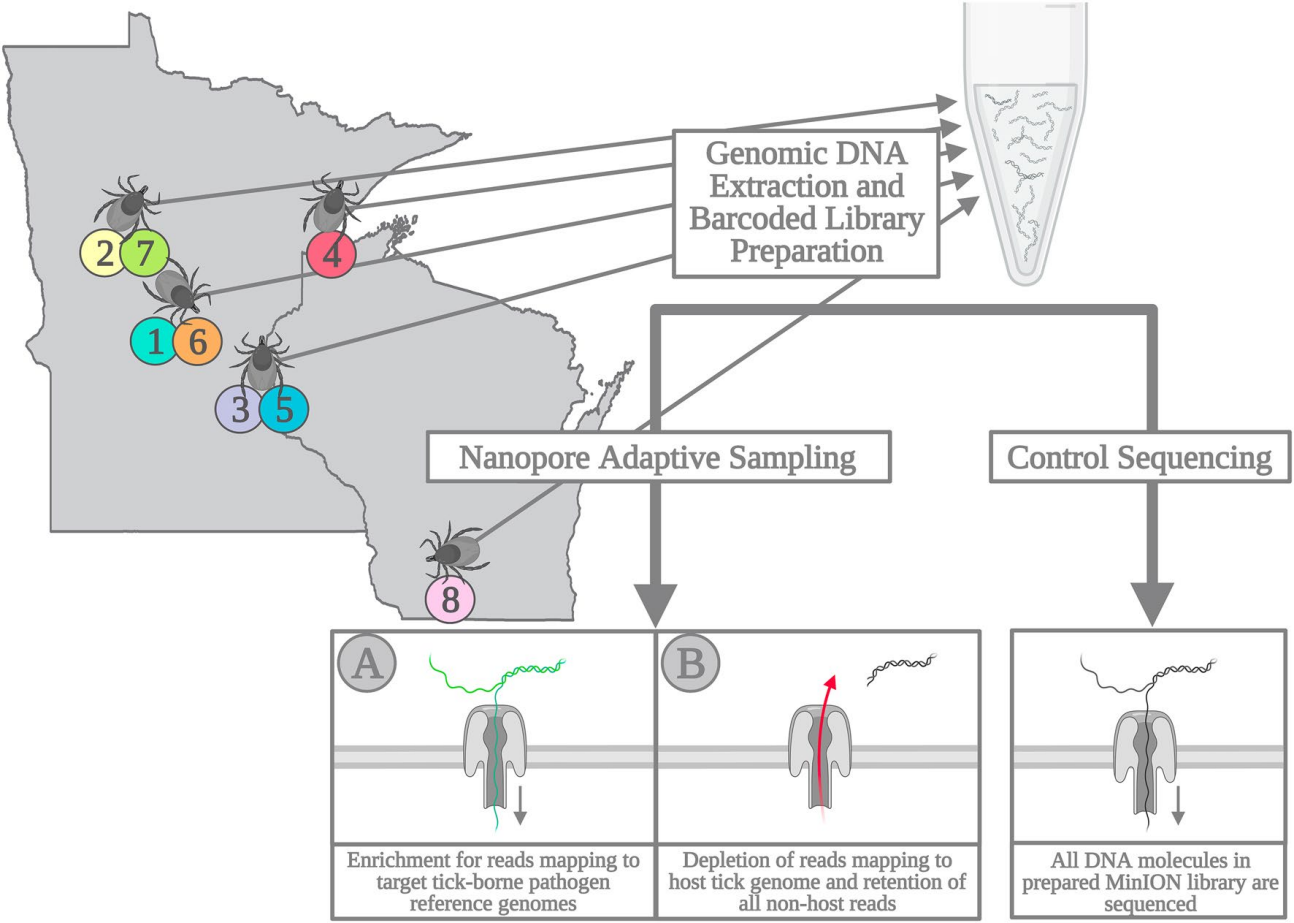
Comparison of nanopore adaptive sampling (NAS) and control sequencing methods. During NAS, real-time mapping of reads against a user-specified reference allows investigators to target desired genomic regions for sequencing enrichment or depletion. Here, we used select whole genome assemblies to enrich for a select assemblage of tick-borne pathogens (**A**), resulting in the rejection of unmapped, non-target reads from the sequencing nanopore. Alternatively, we used the genome assembly of the *I. scapularis* tick vector for a depletion approach in which all mapped host DNA sequences were rejected (**B**), leading to the retention of any unmapped, non-host reads. A control sequencing experiment was also performed without targeted enrichment or depletion and in which all molecules present in the library were sequenced to completion. Map on the left depicts sampling localities in Minnesota and Wisconsin, USA where tick samples originated. Numbers beneath each locality correspond to tick sample numbers referred to throughout the manuscript. Map created using ArcMap software (v10.8.2, ESRI, Redlands, United States).

Nanopore adaptive sampling (NAS) is a recently developed and novel strategy that performs selective sequencing of individual DNA, cDNA, or RNA molecules in real-time using ONT sequencing technology^23,24^. NAS operates through an advanced bioinformatic pipeline that compares nucleotides of individual molecules (~ 200–400 base pairs) against a user-specified reference file every ~ 0.4 s, as sequencing is occurring. This dynamic method has a wide number of applications and can selectively enrich targets of interest (e.g., select genes, chromosomes, mitogenomes, RNAs, etc.) and reject unwanted molecules throughout a given sequencing run^23,25^. NAS can be leveraged for real-time pathogen surveillance and discovery using two approaches. First, depletion of host genomic DNA or RNA sequences can be accomplished in real-time by supplying a host reference genome (e.g., human, bat, rodent, primate, mosquito, tick) and rejecting host reads (Fig. 1B). The resulting sequence data is enriched for the entire metagenomic community and then mined for de novo surveillance of putative pathogens. Second, a reference file containing whole genomes or select genes (e.g., phylogenetically informative loci, antimicrobial resistance genes) of all known pathogens of interest (including both viral and bacterial targets in a single file) can be provided (Fig. 1A). If present within a sample, sequences with a minimum of ~ 70% sequence similarity to the reference will be retained or rejected as specified by the user^23^. This aspect of NAS distinguishes it from all other sequencing methods, as the process can detect extremely small proportions of non-host DNA/RNA (e.g., low abundance viral and/or bacterial pathogens intermixed with > 99% host molecules)^24,26^. A critical observation with respect to NAS is that nanopore-based sequencing routinely produces DNA/RNA reads thousands of bases long. Thus, the metagenomic data generated by NAS increases confidence in resulting surveillance findings and taxonomic identification as entire viral or bacterial genomes can be recovered. For these reasons, NAS-based surveillance can potentially be used for de novo identification of pathogens via unbiased metagenomic/metatranscriptomic enrichment or through the capture of novel strains based on sequence similarity shared between suspected agents and those for which genomic resources are available.

To evaluate the utility of NAS for pathogen surveillance, we attempted to detect and characterize tick-borne pathogens (TBPs) vectored by the blacklegged tick, *Ixodes scapularis* (Acari: Ixodidae). Globally, ixodid ticks include some of the most significant disease vectors of importance to both human and animal health, and are capable of transmitting a wide diversity of bacterial, protozoal, and viral pathogens to their vertebrate hosts. As a group, TBPs serve as favorable targets to understand how NAS can be used for sequence-based pathogen surveillance. Recent studies have highlighted the complex bacterial, viral, and eukaryotic metagenomic communities maintained by tick species and populations, which harbor an abundance of both pathogenic and non-pathogenic microorganisms^22,27–31^. Frequently, individual ticks may be co-infected with multiple TBPs, the epidemiological and clinical consequences of which are still poorly understood^27,32,33^. In the United States, *I. scapularis* represents the vector species of greatest public health importance^34^. While *I. scapularis* is best known for its role in the transmission of *Borrelia burgdorferi* s.l.—the causative agent of Lyme disease—it is increasingly evident that the species is also responsible for transmitting numerous other TBPs. In addition to *B. burgdorferi* s.l., at least six other human pathogens are currently recognized to be vectored by *I. scapularis*, including the causative agents of *Borrelia miyamotoi* disease (caused by a distinct spirochete in the relapsing fever clade), anaplasmosis, ehrlichiosis, babesiosis, and Powassan virus encephalitis^34–37^. A complicating factor in the control of tick-borne diseases has been the dramatic expansion of *I. scapularis* populations over the past decades, which has coincided with increasing *I. scapularis* vector densities and rates of human-tick contact throughout many regions of North America^34,38–40^. As a result, the reported incidence of tick-borne diseases in the U.S. over the past two decades has more than doubled^38^. Despite these trends, current methods for TBP surveillance are limited in their reliance on conventional PCR and ability to detect only a single or limited range of TBPs. To evaluate the potential of nanopore sequencing—in combination with the NAS strategy—for TBP surveillance, we attempted to detect and characterize TBPs from field-collected *I. scapularis* ticks from Minnesota and Wisconsin, USA, two states with a high burden of tick-borne disease^27,28,36,41,42^.

In this work, we leveraged NAS for the detection of four bacterial TBPs: *B. burgdorferi* s.s.; *B. miyamotoi*; *Anaplasma phagocytophilum*; and *Ehrlichia muris eauclairensis* circulating in wild-caught *I. scapularis*. We demonstrate that the NAS method successfully recovered TBP-derived sequences using both enrichment and host depletion strategies, resulting in the sequencing of entire bacterial pathogen genomes. Importantly, our NAS results regarding pathogen status were in full agreement with independently generated Illumina 16S microbiome data. Additionally, adaptive sampling through both enrichment and depletion approaches allowed for clear differentiation between closely related bacterial TBPs (e.g., the Lyme disease agents *B. burgdorferi* s.s. and *B. mayonii*, and the relapsing fever agent *B. miyamotoi*), highlighting its potential utility as a novel approach for metagenomic pathogen surveillance across a variety of arthropod vector species.

## Results

### Tick sampling and sequencing strategies

We isolated total genomic DNA from eight adult female *I. scapularis* specimens collected at different localities across Minnesota and Wisconsin, USA (Fig. 1). The V4 region of the bacterial 16S rRNA gene was amplified across all eight samples, and microbiome sequencing using the Illumina MiSeq platform revealed that all eight ticks were infected with *Borrelia (Borreliella) burgdorferi* s.l. Five of the eight ticks exhibited evidence of co-infection with an additional bacterial tick-borne agent. In total, our 16S Illumina sequencing suggested the likely presence of four bacterial TBPs: *A. phagocytophilum*, *B. burgdorferi* s.l., *B. miyamotoi*, and *E. muris eauclairensis* across our *I. scapularis* samples (Table 1). The total number of classified 16S reads varied by sample and TBP—ranging from 1097 to 34,489—suggesting wide variability in the relative abundance of each bacterial pathogen in its respective tick host.

**Table 1:** Bacterial tick-borne pathogens detected in adult female *I. scapularis* ticks through 16S microbiome sequencing.

	*Anaplasma* spp.	*Borrelia* (*Borrelia*) spp. (Relapsing Fever Group)	*Borrelia* (*Borreliella*) spp. (Lyme Disease Group)	*Ehrlichia* spp.
Tick 1	ND* (1)	ND	Yes (9633)	Yes (34,489)
Tick 2	ND	ND	Yes (21,790)	ND* (1)
Tick 3	ND	Yes (9715)	Yes (1182)	ND
Tick 4	Yes (6410)	ND	Yes (1280)	ND
Tick 5	Yes (5828)	ND	Yes (1097)	ND
Tick 6	Yes (4492)	ND	Yes (3861)	Yes (2,777)
Tick 7	ND	ND	Yes (11,490)	ND* (1)
Tick 8	ND	ND	Yes (18,351)	ND

Numbers in parentheses indicate the number of assigned 16S Illumina reads for each tick-borne pathogen taxa. Reads assigned at the genus level under the Relapsing Fever Group spirochetes Borrelia (Borellia) suggest presence of the I. scapularis-transmitted agent B. miyamotoi, while reads classified under the Lyme Disease Group, Borrelia (Borreliella), are suggestive of tick infection with a member of the B. burgdorferi s.l. clade. ND—not detected; *Taxa for which a single 16S read was assigned are considered likely the result of read misclassification and not indicative of tick infection.

Next, barcoded nanopore libraries were generated using genomic DNA from the same eight *I. scapularis* ticks. We first set out to evaluate the performance of the NAS enrichment approach (Fig. 1A) for targeted TBP detection and prepared two identical nanopore sequencing libraries. In these libraries, four samples were barcoded (Tick 1–Tick 4), pooled, and nanopore sequencing adapters were ligated; both final sequencing libraries contained between 140 and 150 ng of DNA. The paired libraries were then sequenced on MinION R9.4 flow cells over a 24-h runtime. During the sequencing of one library, NAS was enabled through a reference file containing whole genome (and plasmid) sequences of both established and unlikely *Ixodes*-transmitted TBPs was provided for adaptive sampling enrichment (Supplementary Table S1). The second of the paired libraries was sequenced without NAS as a control. The library sequenced with adaptive sampling yielded a total of over 5.5 million reads and 1.9 Gb of sequence data. A comparable amount of total sequencing output was obtained from the control library, with 1.8 Gb of sequence data generated (Table 2). A notably shorter average read length was obtained from the NAS enrichment sequencing experiment in comparison to the control (359.0 bp and 627.3 bp, respectively). This difference in average read length was anticipated and likely due to the retention of a significant number of unmapped *I. scapularis* reads in which the initial 200–400 bp were sequenced prior to being rejected from the nanopore. Overall, both flow cell performance and total sequencing output were comparable between paired NAS enrichment and control sequencing experiments; however, a steeper decrease in active nanopores was noted during NAS enrichment, likely due to increased pore stress caused by the frequent rejection of unmapped DNA molecules (Supplementary Fig. S2–S5).

**Table 2:** Total sequencing output by tick sample generated during NAS and control sequencing experiments.

	Total reads	Total Bases sequenced (Mb)	Read length N50 (bp)	Mean read quality
NAS enrichment				
Tick 1	1,154,447	421.4	397	9.9
Tick 2	1,115,550	390.2	376	9.9
Tick 3	644,767	233.8	400	10
Tick 4	1,853,677	662.8	386	9.9
Total	4,768,441	1708.2	389	9.8
Control				
Tick 1	579,959	331.7	771	10
Tick 2	606,365	302.3	605	10.1
Tick 3	360,105	226.8	1,005	10.1
Tick 4	959,027	654.8	1,185	10.1
Total	2,505,456	1515.7	888	10.1
NAS depletion				
Tick 5	2,233,967	621.3	328	13.7
Tick 6	1,494,667	392.9	317	13.6
Tick 7	712,929	186.5	312	13.6
Tick 8	837,779	247.5	348	13.5
Total	5,279,342	1448.1	327	13.5

Total sequencing output and summary statistics for *Ixodes scapularis* tick samples used for both NAS enrichment, NAS depletion, and control MinION experiments.

### Evaluation of NAS with enrichment for tick-borne pathogens

**Figure 2:**
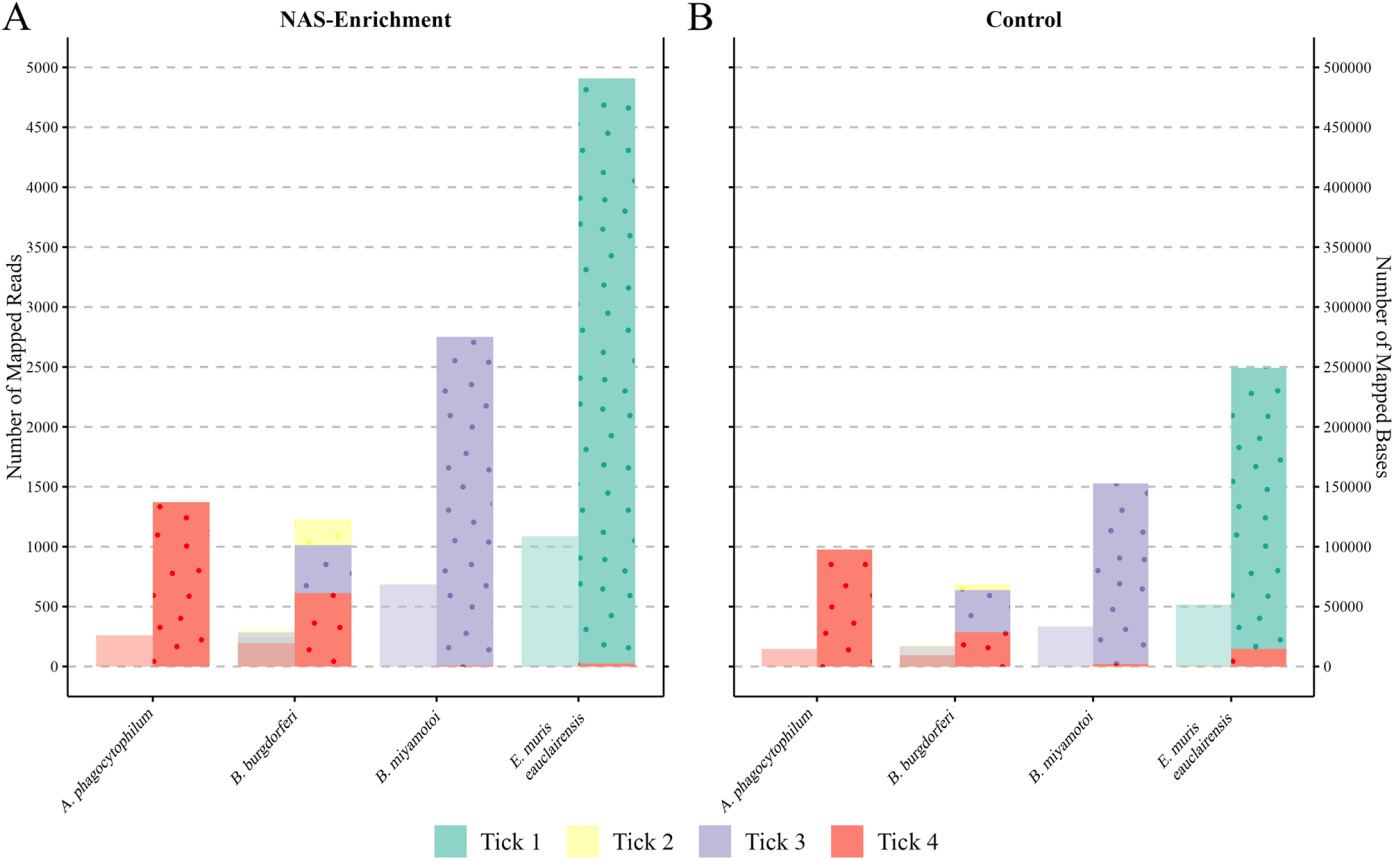
Enrichment through adaptive sampling yields an increased number of total reads and bases mapping to target tick-borne pathogen taxa. Genomic DNA from individual *I. scapularis* samples (Ticks 1–4) was sequenced during NAS enrichment and control experiments. (**A**) Total reads and corresponding bases successfully mapping to each target tick-borne pathogen through NAS enrichment; number of reads are shown in the left columns and total bases shown in right (dotted columns) for each detected tick-borne pathogen genome. (**B**) Mapped reads (left) and bases (right, dotted columns) generated through unenriched control sequencing. Across each of the four bacterial pathogens detected in these paired NAS and control experiments, adaptive sampling achieved roughly two-fold read and base enrichment in comparison to control sequencing.

Reads were basecalled in real-time using the fast basecalling model in guppy (i.e., basecalling speed is prioritized over read accuracy). Real-time basecalling is a notable requirement for NAS, as rapidly basecalled reads are needed for alignment to the reference sequences used for either enrichment or depletion. In total, our paired NAS enrichment and control libraries yielded a total of 4216 and 2631 successfully mapped reads, respectively. The real-time mapping results we obtained were in full agreement with our previous 16S Illumina MiSeq findings in terms of TBPs detected by sample. Importantly, real-time alignment also enabled us to distinguish between the closely-related *B. burgdorferi* s.l. Lyme Disease Group spirochetes (i.e., *B. burgdorferi* s.s. and *B. mayonii*) to confirm that all four ticks were infected with *B. burgdorferi* s.s., a level of taxonomic resolution not attainable from our 16S Illumina MiSeq data. Following the conclusion of each sequencing experiment, we conducted post-hoc basecalling of raw nanopore Fast5 signal data using a high accuracy basecalling model to improve the per-base accuracy of generated reads. After quality filtering and extraction of reads mapping to our target TBPs, we observed that NAS achieved roughly two-fold total read enrichment over our control sequencing library (Fig. 2). This roughly two-fold enrichment was observed to be consistent across all four tick samples and individual TBPs sequenced over the NAS enrichment experiment. Similarly, the total number of successfully mapped bases indicated a similar level of sequencing enrichment when NAS was enabled in comparison to our control (Fig. 2). During these analyses, we also noted that numerous reads for both sequencing runs mapped to the genome of the eukaryotic pathogen, *Babesia microti*; however, further characterization of these reads suggested that they derived from repetitive elements within the *I. scapularis* genome and did not suggest active infection. For this reason, we chose to focus our remaining analyses on characterizing bacterial TBPs only.

Total reads were also processed using the kraken2 package, an alternative minimizer-based classification tool independent of our previous mapping and characterization using the minimap2 package for real-time TBP genome alignment^43,44^. This approach for read classification provided an additional and independent assessment of taxonomic diversity found within each *I. scapularis* sample. The output from these analyses supported our previous findings in terms of which bacterial TBPs were detected and the level of sequencing enrichment achieved using NAS (Table 3). Overall, the relative numbers of nanopore reads classified using both the minimap2 approach and kraken2 were comparable and consistent between samples and sequencing experiments. The kraken2 pipeline was also used to provide additional confirmation of the presence of *B. burgdorferi* s.s. in our samples. Notably few reads from our samples were classified as *B. mayonii*, while > 100 reads were classified as *B. burgdorferi* s.s.

**Table 3:** Taxonomic classification results for reads generated during NAS and control experiments.

	Bacterial tick-borne pathogen genome							
	*A. phagocytophilum*	*B. burgdorferi* s.s	*B. miyamotoi*	*E. muris eauclairensis*	*A. phagocytophilum*	*B. burgdorferi* s.s	*B. miyamotoi*	*E. muris eauclairensis*
	NAS enrichment				Control			
Tick 1	0	274(2.4)	0	769(2.1)	1	113	0	364
Tick 2	0	351(1.8)	5	0	0	194	4	1
Tick 3	0	334(1.8)	1113(2.1)	2	1	190	523	2
Tick 4	238(1.8)	218(1.7)	5	2	136	127	2	1
	NAS depletion							
Tick 5	153	657	0	0				
Tick 6	665	1457	1	146				
Tick 7	4	190	0	0				
Tick 8	14	422	1	0				

Numbers presented under each TBP taxonomic group indicate the number of ONT reads successfully identified taxonomy at the species level using the kraken2 metagenomic classification pipeline. Numbers within parentheses denote the fold enrichment achieved in classified reads between ticks sequenced during paired adaptive sampling and control experiments.

**Figure 3:**
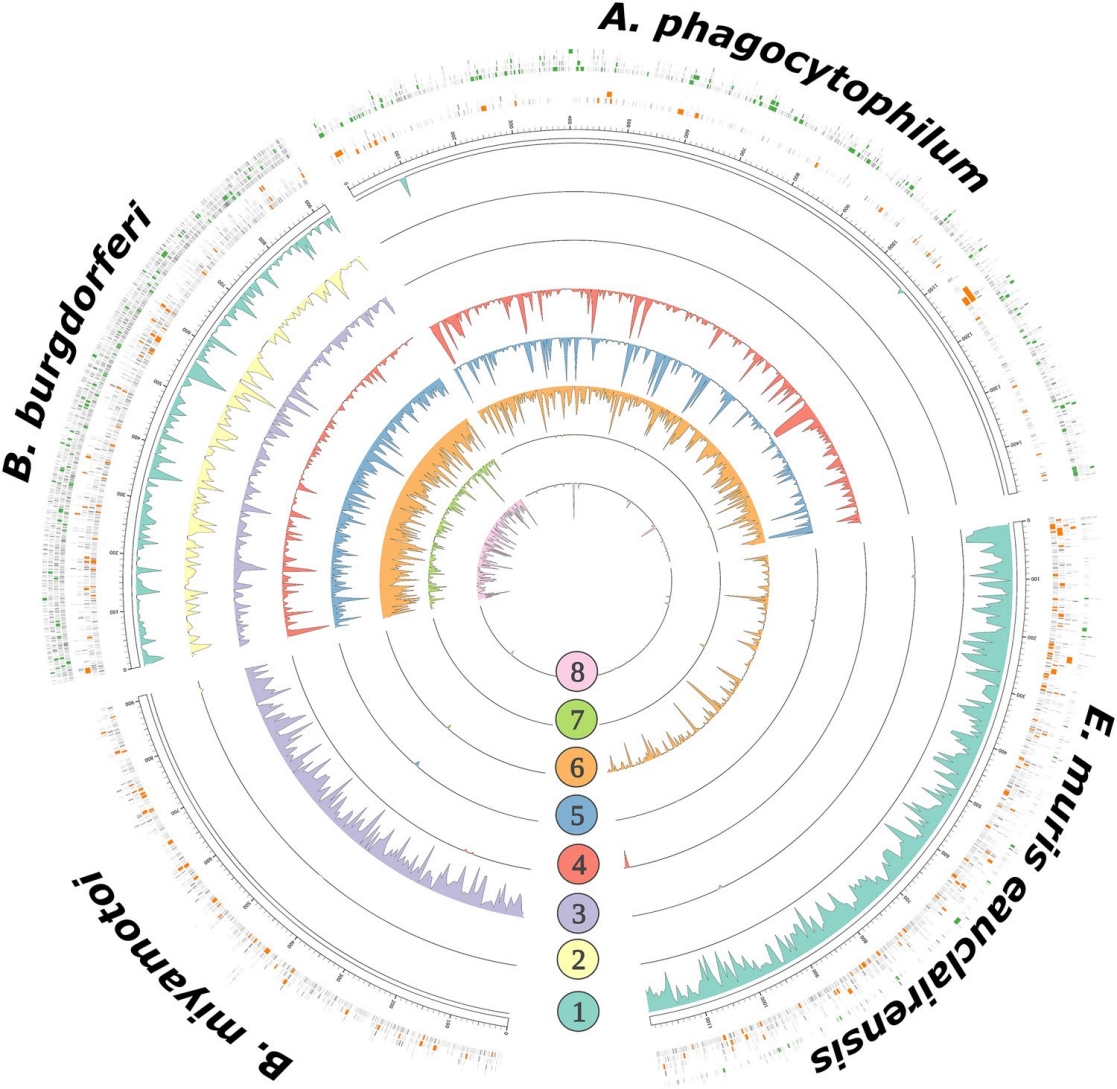
Recovered metagenomic data generated through NAS by both enrichment and depletion strategies span whole genome assemblies of bacterial tick-borne pathogens. Circos plot depticting complete genomes of four bacterial TBPs: *B. burgdorferi* s.s., *A. phagocytophilum*, *E. muris eauclairensis*, and *B. miyamotoi*. Outermost bands are individual ONT reads mapping to each bacterial genome. Reads > 2 kb in length generated during NAS enrichment (Tick 1–Tick 4) are visualized in orange, and > 2 kb reads generated during NAS depletion (Tick 5–Tick 8) are shown in green. Inner track histograms depict mean coverage for mapped bases across pathogen genomes for individual tick samples calculated over a sliding widow of 5 kb.

After concluding that sequencing via NAS achieved a notable level of TBP sequence enrichment over our control, we assessed where reads mapped to corresponding bacterial TBP genomes. We extracted NAS mapping coordinates for all individual mapped reads and then plotted these across the complete genomes of the corresponding TBPs (Fig. 3). These findings demonstrate that sequencing via NAS resulted in a substantial amount of long-read sequence data with numerous bacterial TBP sequences in excess of 5 kb. Additionally, we demonstrate that pathogen-derived NAS sequences spanned the assembled genomes, at varying depths of coverage, of each TBP.

### Performance of NAS using a host depletion approach

**Figure 4:**
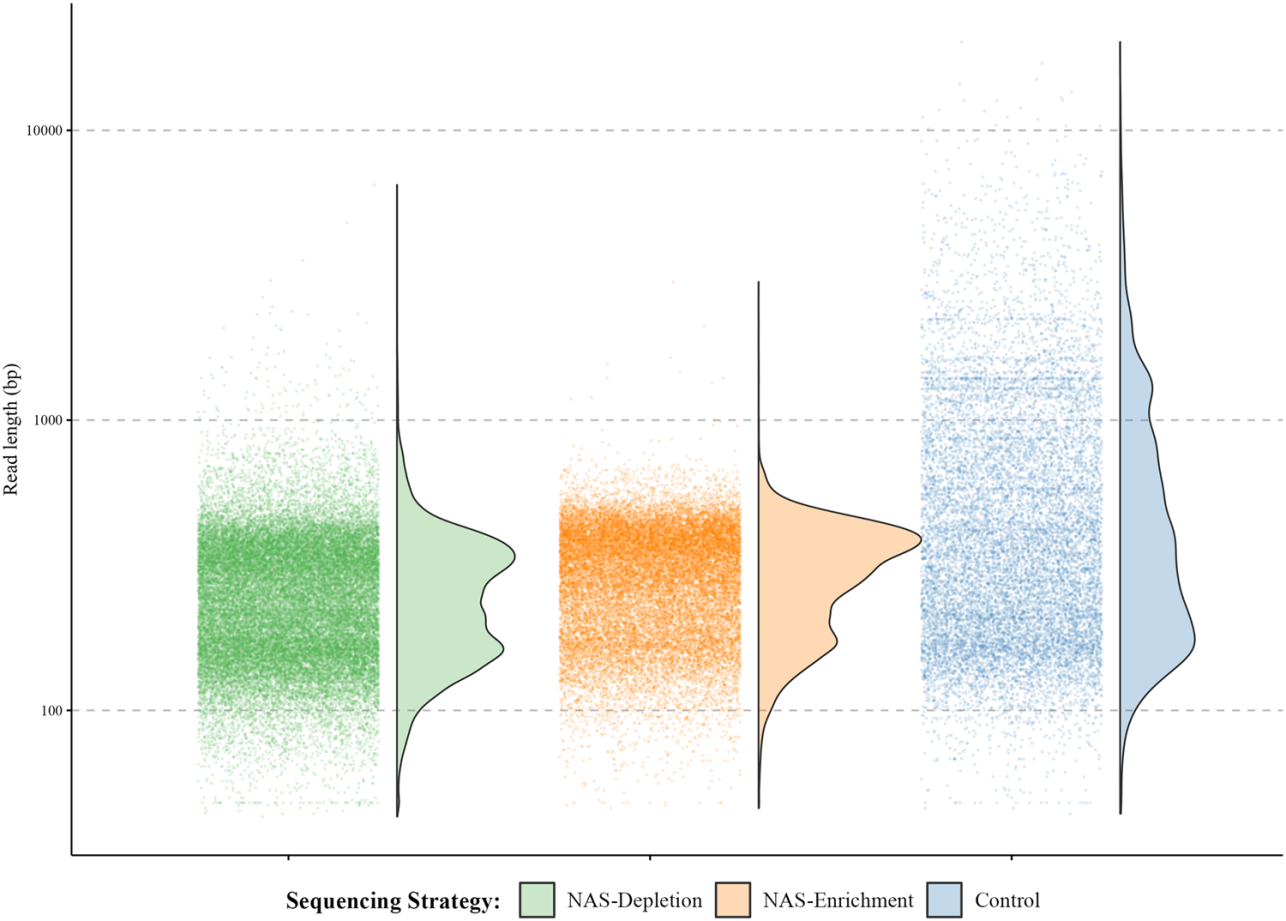
Distribution of reads mapping against the non-target bacterial *I. scapularis* endosymbiont, *R. buchneri*. Total *R. buchneri-*mapped reads generated over NAS depletion, NAS enrichment, and control sequencing experiments are presented as points by read length over a logarithmic scale. Over both NAS-depletion (green) and NAS-enrichment (orange) sequencing strategies, the read length distribution shows a greater proportion of reads < 1000 bp, indicative of real-time rejection of non-target *R. buchneri* reads during each sequencing experiment. Conversely, during the control experiment (blue), an increased proportion of reads between 1000–10,000 bp is observed, as all non-target *R. buchneri* reads present in the library were sequenced fully.

To compare the efficacy of NAS for both enrichment of pathogen targets and depletion of host genomic DNA, an additional library containing four additional *I. scapularis* ticks (Tick 5—Tick 8) was produced and sequenced. As above, sequencing was done using a new MinION R9.4 flow cell and post-hoc basecalling of raw signal data was conducted using a high accuracy basecalling model. During sequencing of this library, adaptive sampling was enabled with specifications for the depletion of the complete *I. scapularis* genome (Fig. 1B). In addition to the host tick genome, the whole genome of *Rickettsia buchneri* was also provided for depletion, as this non-pathogenic bacterial endosymbiont is frequently found at an overwhelming abundance in *I. scapularis* ticks^45,46^. Overall, we observed similar output measurements following this depletion experiment as was seen during NAS enrichment (Table 2). Across all ticks sequenced through NAS depletion, a short average read length was again observed, as an abundance of reads which successfully mapped to the *I. scapularis* or *R. buchneri* genomes were rejected from the sequencing nanopores and in which the first few hundred base pairs of data were retained. Following a similar approach as was taken for our NAS enrichment and control experiments, reads from each tick generated during this depletion run were characterized using minimap2 and the kraken2 packages. Across these four ticks sequenced by NAS depletion, we again achieved full concordance with our Illumina 16S data in terms of which TBP taxa were detected (Table 3). In addition, we noted numerous similarities between our NAS enrichment and depletion experiments. Although the total number of reads that were successfully classified to a particular TBP showed greater variability in our NAS depletion experiment, both resulted in similar numbers of successfully mapped and classified reads and showed similar success at differentiating between related TBPs (i.e., classification of reads clearly demonstrated that ticks were infected with *B. burgdorferi* s.s. and not *B. mayonii* or *B. miyamotoi*) (Fig. 3). In comparison to the control, data generated over both NAS enrichment and depletion demonstrated substantially fewer long-read data mapping against the bacterial endosymbiont, *R. buchneri*, suggesting that either NAS approaches performed comparably at depleting sequencing output for this non-target organism (Fig. 4).

## Discussion

Surveillance of vector arthropods for pathogens is an important part of the public health repertoire for combating vector-borne diseases. The U.S. Centers for Disease Control and Prevention have stated that “new tools for preventing tick-borne diseases are urgently needed…”^47^. Nanopore sequencing allows for rapid detection of pathogens with minimal sample preparation and reagent requirements, and can be performed across a variety of field conditions. This facet of the technology brings the possibility of arthropod-borne pathogen surveillance sequencing to groups and regions without ready access to laboratory or internet resources. In places with high rates of endemic vector-borne disease, local public health organizations can use nanopore sequencing to rapidly determine the prevalence of pathogens in vector species without needing to rely on the testing facilities of state or national laboratories. Moreover, unlike traditional PCR which allows for pathogen detection by amplifying short regions of template DNA, nanopore sequencing produces long reads that provide substantially more genomic information. As such, nanopore sequencing will see utility in the differentiation of pathogenic and non-pathogenic strains within the same species. For example, human infectious *A. phagocytophilum* strains can be rapidly differentiated from non-human infectious ungulate strains without requiring nested PCR^48^. Genetically similar species, such as spotted fever group rickettsiae (some of which are not human infectious), will be rapidly differentiable in the field rather than relying on PCR amplification followed by sequencing or restriction fragment length polymorphism techniques, as is currently done^49,50^.

The recent development of adaptive sampling on ONT sequencing platforms offers even greater potential for sequence-based applications in arthropod vector surveillance. NAS can be used to selectively sequence DNA or RNA molecules associated with entire pathogen genomes via NAS enrichment. Using NAS for such a targeted enrichment approach, investigators can target a wide range of pathogen-derived nucleic acids which may be substantially less abundant in their library in comparison to those of either the arthropod host or other microbes. Using NAS, investigators can also choose to rapidly deplete arthropod host-associated sequences to generate sequence data for entire metagenomic communities and any vector-borne pathogens contained therein. As we demonstrate here, NAS can also be leveraged for the simultaneous depletion of both host reads and those of abundant non-target microbes. Many tick species contain large quantities of symbiotic bacteria belonging to the same genera as TBPs such as *Rickettsia amblyommatis* in *Amblyomma* spp. or *Francisella* spp*.* in *Dermacentor* spp. In *I. scapularis*, 10^6^–10^7^ *Rickettsia buchneri* may be present in a single female^51^ and this symbiont commonly represents > 80% of 16S sequences in microbiome studies of female ticks^45,46,52^. The sheer quantity of genetic material from these bacteria can overwhelm less-abundant or rare sequences in methods used to evaluate the presence of multiple bacterial species within field sampled ticks. Thus, NAS via either genomic enrichment of pathogen targets or depletion of non-target organisms provide a valuable means to prevent output of unwanted sequence data.

We utilized nanopore MinION sequencing in combination with NAS to detect and molecularly characterize four bacterial TBPs (*B. burgdorferi s.s.*; *B. miyamotoi*; *A. phagocytophilum*; and *E. muris eauclairensis*) associated with eight female *I. scapularis* ticks. By enriching for whole genome assemblies of these pathogens, we confirmed that NAS was successful at targeting each detected TBP, resulting in an approximately two-fold enrichment of recovered single-molecule sequences over our control sequencing experiment (Fig. 2; Supplementary Fig. S1). Using an alternate approach in which we used NAS to deplete our library for reads derived from both *I. scapularis* and *R. buchneri*, we were able to similarly detect TBP infections in individual tick samples. Importantly, this depletion approach has the potential advantage of being able to gather an "unbiased" view of metagenomic diversity across a variety of arthropod taxa for use in vector-borne pathogen discovery applications in which the species/strain of pathogen targeted may be unknown or poorly understood.

Our findings from both NAS enrichment and depletion experiments were supported by 16S Illumina microbiome sequencing data and an independent bioinformatic pipeline for classifying our nanopore reads. Importantly, nanopore sequencing enabled us to achieve a high level of taxonomic resolution in these samples to conclude that all eight ticks we sequenced were infected with *B. burgdorferi s.s.* and not the closely related species Lyme disease group spirochete *B. mayonii*. As two closely-related *I. scapularis*-transmitted pathogens, the Lyme disease agents *B. burgdorferi s.s.* and *B. mayonii* are separated by only ~ 5% genetic distance^53^. Despite their relatedness, our analyses and taxonomic classification of reads obtained by NAS were able to reliably differentiate between these bacterial species. Mapping of our long-read data against both bacterial pathogen genomes and classifying NAS reads individually using kraken2 both clearly suggested that *B. burgdorferi s.s.* was the sole *Borrelia* species present in our samples. Although PCR-based TBP surveillance approaches can easily provide similar species-level resolution, the NAS approach enabled us to generate large amounts of sequence data spanning entire bacterial TBP genomes. Using NAS, we noted the recovery of extremely long, intact DNA sequences associated with each bacterial TBP. For example, we received a single *A. phagocytophilum* read in excess of 26 kb, in addition to numerous > 5 kb reads across all detected TBPs (Fig. 3). Such long sequence lengths of individual DNA molecules secured using the NAS bioinformatic pipeline opens the door to genome-scale pathogen surveillance, an opportunity that is not easily explored using traditional methods.

Despite documenting the functionality of NAS for TBP research, we noted that numerous nanopore reads incorrectly mapped against the *Ba. microti* genome, a protozoal eukaryotic pathogen. Additional analysis found that these reads contained repetitive DNA sequences derived from the host *Ixodes* tick. Thus, we recommend caution when interpreting NAS sequence data wherein highly conserved or repetitive sequences have the potential to provide incorrect taxonomic assignment or spurious results. In addition, although our two-fold sequence enrichment provided considerable genomic information for targeted low-abundance TBPs, we also note that increased levels of enrichment are likely possible through a combination of extracting higher molecular weight genomic DNA and the loading of a larger sequencing library onto the MinION flow cell. Conversely, while sequencing at the level of the individual tick can provide detailed information about pathogen presence, microbial diversity and co-occurrence relationships^27,29^, it is likely not feasible nor cost effective for robust surveillance in many settings (e.g., an environment where pathogens are at very low prevalence). Fortunately, NAS-based surveillance approaches are widely scalable and can be adapted for use with pooled samples. These caveats notwithstanding, nanopore sequencing in combination with adaptive sampling showed considerable promise at detecting bacterial TBPs using unprocessed genomic DNA and limited sample processing steps. As "proof-of-concept", our study assessed eight field-collected *I. scapularis* ticks using the MinION sequencing platform; however, we anticipate that with optimization of library preparation methods and performance improvements to the NAS pipeline, a substantially greater number of samples may be multiplexed on a single flow cell. We predict that, as technologies and computational analyses associated with nanopore sequencing and adaptive sampling improve, a multitude of exciting applications for real-time metagenomic pathogen biosurveillance will emerge, thus facilitating unprecedented insights into host–pathogen systems.

## Methods

### Tick sample collection and nucleic acid extraction.

As part of larger sampling efforts, ticks were collected using a standard cloth dragging technique from five localities in Minnesota and Wisconsin between April 2017 and June 2019. Sampling consisted of dragging a light-colored 1 m^2^ cloth over the ground and low-laying vegetation to attract host-seeking ticks. Ticks attached to the drag cloth were removed using forceps and visually identified to species, developmental stage, and sex. Adult female *I. scapularis* specimens were selected from each locality sampled to be included in this study. Surface contaminants were removed by sequentially washing ticks in Tween 20 and 0.5% benzalkonium chloride solutions; ticks were next rinsed with distilled water and 70% ethanol, and stored in fresh 70% ethanol prior to nucleic acid extraction. To promote effective tissue lysis, whole ticks were bisected using a sterile 16-gauge needle and incubated in a lysis buffer solution (i.e., 180 μl Qiagen Buffer ATL and 20 μl proteinase K) at 56 °C overnight. The resulting digestion mixture was processed using a DNeasy Blood and Tissue Kit (Qiagen, Hilden, Germany) following manufacturer instructions. DNA extracts were quantified using a Qubit 4 fluorometer (Invitrogen, Carlsbad, United States) and visualized by agarose gel electrophoresis.

### 16S microbiome sequencing.

Characterization of microbial diversity was accomplished through amplification of the V4 region of the 16S rRNA gene using the Meta_V4_515F (TCG-TCG-GCA-GCG-TCA-GAT-GTG-TAT-AAG-AGA-CAG-GTG-CCA-GCM-GCC-GCG-GTA-A) and Meta_V4_806R (GTCTCGTGGGCTCGGAGATGTGTATAAGAGACAGGGACTACHVGGGTWTCTAAT) primers. An initial qPCR step was performed using the Meta_V4_515F and Meta_V4_806R primer pair, and consisted of an initial 95° C denaturation for five minutes, 35 cycles of 98° C denaturation for 20 s, 55° C annealing for 15 s, and 72° C extension for 60 s, and concluded with a final extension for five minutes at 72° C. These data were used to normalize each tick template sample to approximately 167,000 molecules/μl. Next, a conventional PCR using the same primer pair and normalized template was conducted using the following parameters: initial denaturation at 95° C for 5 min; 25 cycles of 98° C denaturation for 20 s, 55° C annealing for 15 s; and 72° C extension for 60 s; followed by a 72° C final extension for 5 min. A 1:100 dilution of the resulting PCR products was done, and 5 μl of this diluted product was used for a second PCR consisting of forward (AATGATACGGCGACCACCGAGATCTACAC[i5]TCGTCGGCAGCGTC) and reverse (CAAGCAGAAGACGGCATACGAGAT[i7]GTCTCGTGGGCTCGG) Illumina indexing primers for Nextera adapters. The specification for this second PCR were as follows: initial denaturation at 95° C for 5 min; 10 cycles of 98° C denaturation for 20 s, 55° C annealing for 15 s; 72° C extension for 60 s; followed by a final 72° C extension for 5 min. Second round PCR products were then pooled, denatured with NaOH, diluted to 8 pM using Illumina HT1 buffer, spiked with 15% PhiX, and denatured at 96° C for 2 min. The prepared Illumina library was sequenced on the Illumina MiSeq system with a 600 cycle v3 kit. The resulting raw Illumina paired-end sequence data are deposited in the National Center for Biotechnology Information (NCBI) Sequence Read Archive (SRA) under BioProject PRJNA790938, BioSamples SAMN24251610 - SAMN24251617.

### Nanopore library preparation.

Genomic DNA libraries were generated following the Sequencing Ligation Kit SQK-LSK109 (ONT) protocol, using between 0.8 and 1.1 μg of total input DNA from each of the eight tick samples. Repair and end-prep of DNA molecules was carried out using NEBNext FFPE DNA Repair and Ultra II End Repair/dA-Tailing modules (New England Biolabs Inc., Ipswich, United States), incubated at 20 °C for 5 min followed by heat-inactivation at 65 °C for an additional 5 min. Samples were purified using AMPure XP beads (Beckman Coulter, Indianapolis, United States) on a magnetic separation rack at a ratio of 1.8:1 beads-to-sample. ONT barcodes were ligated to DNAs for each of the eight tick samples using Blunt/TA Ligase Master Mix (New England Biolabs Inc., Ipswich, United States) and the EXP-NBD104 Native Barcoding Expansion Kit (ONT). Following barcode ligation, samples were quantified on a Qubit 4 fluorometer (Invitrogen, Carlsbad, United States). For four barcoded samples (Tick 1–Tick 4) equimolar amounts of each barcode were pooled and used to generate two identical nanopore libraries for evaluation via NAS enrichment and control sequencing experiments. The additional barcoded samples (Tick 5–Tick 8) were also pooled at roughly equimolar amounts into a separate library for sequencing via NAS depletion, though total genomic DNA recovered from these specimens precluded generation of paired libraries for these samples. For each library, ONT sequencing adapters were ligated using NEBNext Quick Ligation Reaction Buffer/Quick T4 DNA Ligase (New England Biolabs Inc., Ipswich, United States), and ONT Short Fragment Buffer was used during the final library wash to retain fragments of all lengths. Final pooled libraries were eluted in a volume of 15 μl at 37° C, quantified as above, and sequenced immediately or stored briefly at 4° C prior to sequencing.

### MinION sequencing and basecalling

Pooled libraries were sequenced over three separate experiments, with each experiment employing a new FLO-MIN106 flow cell using R9.4 sequencing chemistry. Each flow cell was checked immediately prior to sequencing to ensure that > 1,200 active pores were available for sequencing. Flow cells were primed and loaded on an ONT MinION Mk1B device connected to a custom desktop computer operating on Ubuntu 18.04 LTS and with the following hardware specifications: a GeForce 2080Ti GPU (Nvidia, Santa Clara, United States); a 24-core Ryzen 3900 × CPU (Advanced Micro Devices, Inc., Santa Clara, United States); 64 Gb RAM; a 1 TB SSD; and 15 TB total onboard storage. Sequencing experiments were initiated and monitored using MinKNOW software, v20.10.3. Adaptive sampling to enrich for pathogen-associated reads was carried out for one library using ReadUntil (https://github.com/nanoporetech/read_until_api)^23^, integrated into the MinKNOW platform. To achieve adaptive sampling, reference files in indexed *.mmi* format containing whole genome sequences and coordinates of potential *I. scapularis*-transmitted pathogens were supplied for real-time enrichment; associated plasmid sequences were also included. These pathogens consisted of *B. burgdorferi* s.s.; *B. mayonii*; *B. miyamotoi*; *A. phagocytophilum*; *E. muris eauclairensis; Ba. microti;* Powassan virus, as well as the unlikely *I. scapularis*-transmitted agents *Bartonella henselae* and the mitogenome for *Dirofilaria immitis* (Supplementary Table S1). As a control, the paired library was sequenced without adaptive sampling enrichment. The third sequencing experiment was performed using the NAS depletion approach with Tick 5–Tick 8 (Fig. 1B). For this experiment, the *I.* scapularis genome (NCBI Assembly: GCA_016920785.2) was used as a reference for real-time host depletion with the NAS method. In addition, the complete genome assembly for *R. buchneri* (GCF_000160735.1) was concatenated to the reference to provide supplemental depletion for this highly abundant, non-pathogenic bacterial tick symbiont.

For all libraries, real-time alignment using the TBP enrichment reference file was enabled in MinKNOW to allow mapping of reads against our target TBP genomes through minimap2^44^. Reads initially mapped during sequencing in real-time were basecalled using the fast basecalling model in Guppy. Each sequencing experiments was allowed to proceed for roughly 24 h to allow each library to be sequenced to near completion. For all downstream analyses, raw reads in Fast5 format were re-basecalled and demultiplexed using Guppy, v4.2.2, in GPU mode using the high accuracy (HAC) basecalling model. All raw nanopore sequence data generated herein are available via the NCBI SRA under BioProject ID PRJNA790938; BioSamples SAMN24251598–SAMN24251609.

### Bioinformatic processing

Paired-end raw 16S reads generated on the Illumina MiSeq platform were demultiplexed and Nextera adapters were trimmed. The resulting reads were processed using the DADA2 pipeline (https://github.com/benjjneb/dada2) to taxonomically assign 16S amplicons to the level of the bacterial genus. The resulting amplicon sequence variant file was queried for pathogenic agents of interest (e.g., *Anaplasma*, *Borrelia* (relapsing fever-associated species), *Borreliella* (Lyme disease-associated Borrelia species), *Ehrlichia*) to determine pathogen presence and the relative abundance of raw V4 16S reads for each sample.

For all nanopore sequencing runs, demultiplexed and HAC basecalled FASTQs were concatenated by sample, and passed reads with a mean read quality score of greater than 7 were retained for downstream analyses. Quality statistics from NAS enrichment, NAS depletion, and control nanopore sequencing experiments were assessed using NanoPlot (https://github.com/wdecoster/NanoPlot), NanoComp (https://github.com/wdecoster/nanocomp), and Nanoq software packages^54,55^. Adaptive sampling .csv and sequencing summary .txt output files were queried to assess which nanopore reads from both sequencing experiments successfully aligned to our target TBP genomes. Concatenated FASTQs by each tick sample and sequencing experiment were further processed using minimap2, samtools, and bedtools software packages to assess read mapping and coverage statistics^44,56,57^. Independent taxonomic classification was also performed using the kraken2 package, v2.1.1, (https://github.com/DerrickWood/kraken2)^43^ and the pre-assembled "PlusPF" database (https://benlangmead.github.io/aws-indexes/k2) for assessing archaeal, bacterial, viral, protozoal, and fungal metagenomic diversity.

## Supplementary Information

Supplementary Information.

## Data Availability

All raw data from ONT MinION and Illumina MiSeq sequencing experiments described here are deposited in FASTQ format within the NCBI Sequence Read Archive (SRA) repository under BioProject ID PRJNA790938. Data generated through NAS enrichment, NAS depletion, and control sequencing experiments are accessioned under the following BioSamples: SAMN24251598 - SAMN24251609. BioSample numbers for the corresponding paired-end Illumina MiSeq 16S data are accessioned as follows: SAMN24251610 - SAMN24251617.
