# Supplementary material for: Metagenomic surveillance for bacterial tick-borne pathogens using nanopore adaptive sampling
Source: Sci Rep. 2023 Jul 7;13:10991. doi: 10.1038/s41598-023-37134-9 (PMC10328957; doi:10.1038/s41598-023-37134-9)
Supplement: Supplementary file 1 — Supplementary Information. [file 41598_2023_37134_MOESM1_ESM.pdf]

Supplementary Information

Metagenomic surveillance for bacterial tick-borne pathogens using nanopore adaptive sampling

Evan J. Kipp, Laramie L. Lindsey, Benedict Khoo, Christopher Faulk, Jonathan D. Oliver, Peter A. Larsen

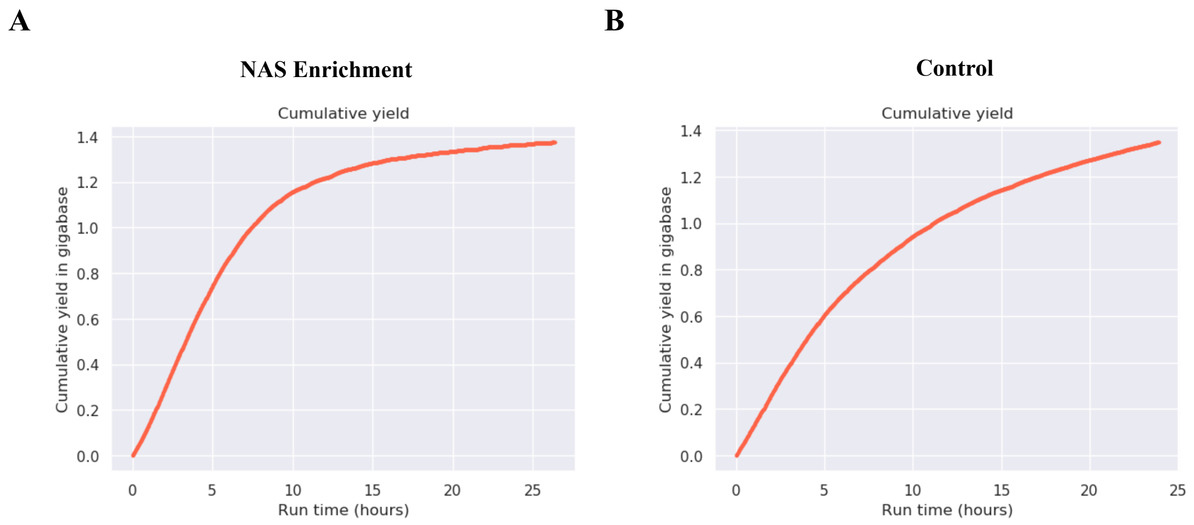

Supplementary Figure 1. Total raw sequencing output by run time over NAS enrichment (A) and control (B) sequencing experiments. Total data generated for each barcoded tick (Tick 1 – Tick 4) were comparable between both libraries sequenced during each experiment.

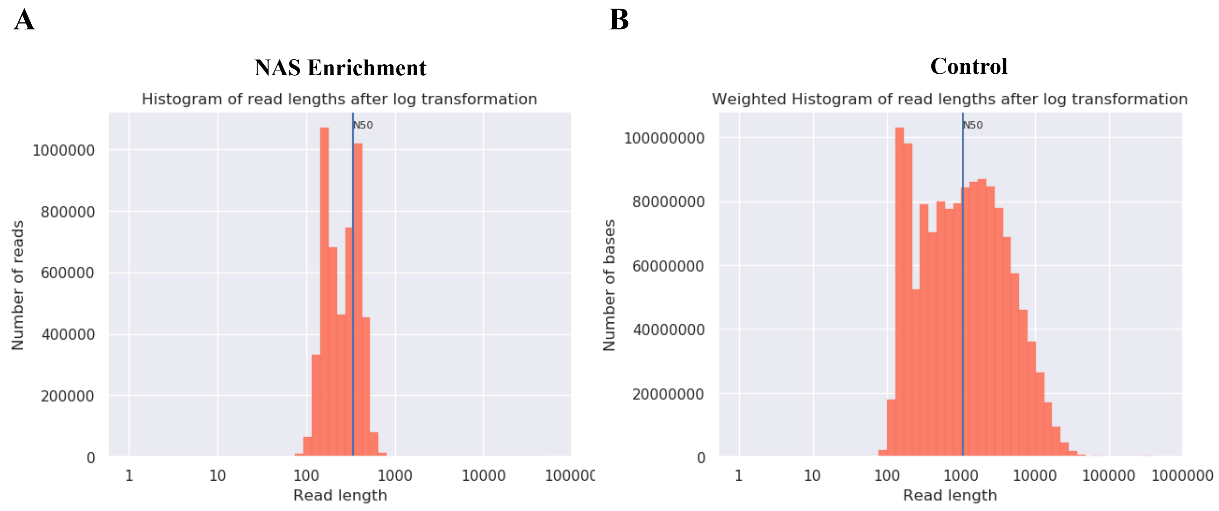

Supplementary Figure 2. Comparison of log-transformed read lengths of raw data generated during paired NAS enrichment (A) and control (B) sequencing experiments. The shorter average read length and N50 seen during NAS enrichment is suggestive of non-target sequences rejected from sequencing during adaptive sampling.

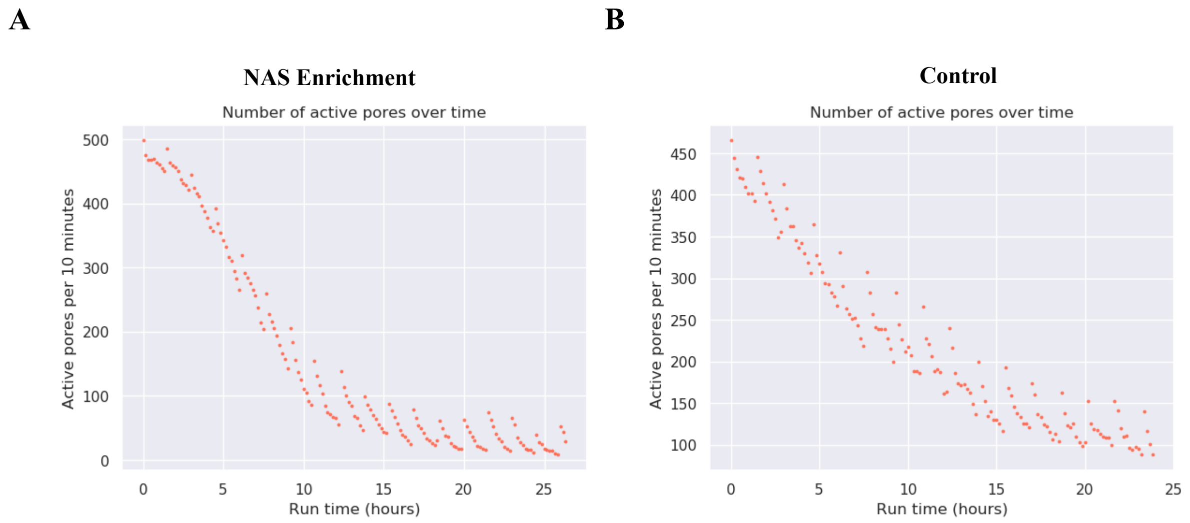

Supplementary Figure 3. Comparison of nanopores available for sequencing by run time for paired NAS enrichment (A) and control (B) sequencing experiments. Note the more rapid decrease in active pores for NAS enrichment run likely caused by pore stress imposed by NAS-based rejection of unmapped, non-target DNA molecules.

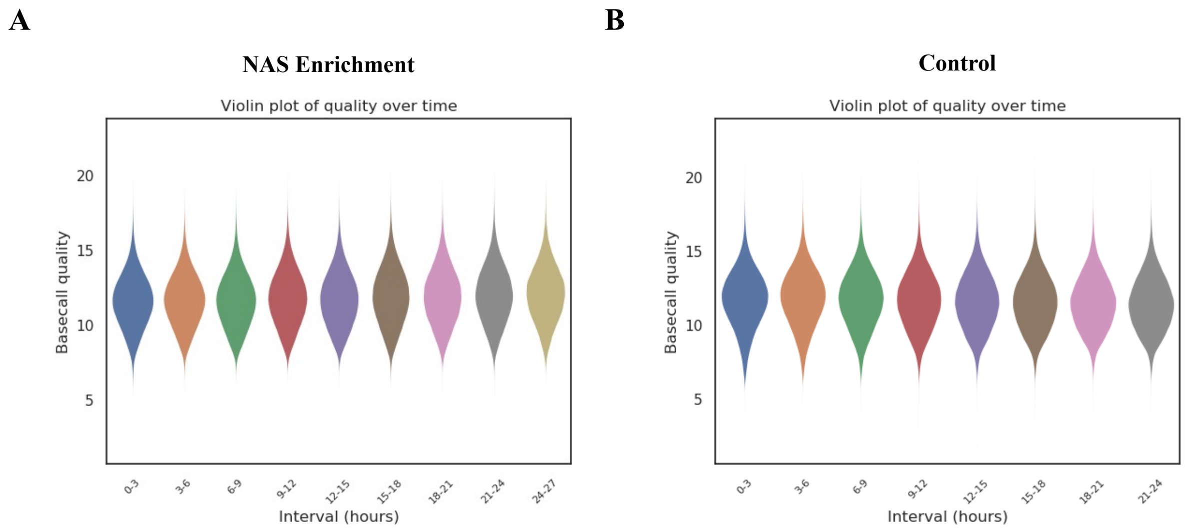

Supplementary Figure 4. Violin plots of basecalling quality scores for paired NAS enrichment (A) and control (B) sequencing experiments by run time. Both sequencing strategies generated reads of similar overall average quality scores.

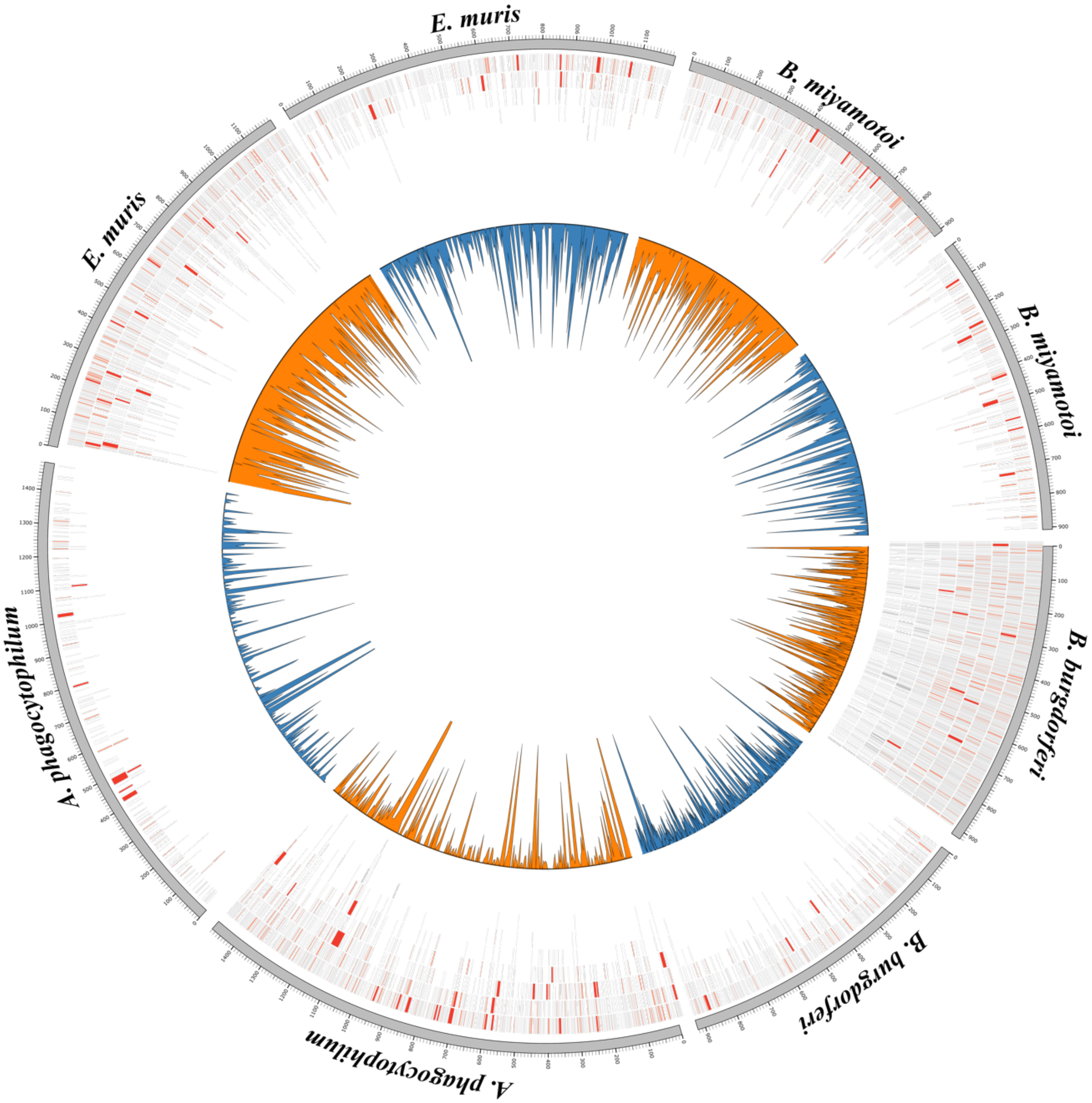

Supplementary Figure 5. Circos plot of mapped data from detected tick-borne pathogen genomes recovered during paired NAS enrichment and control sequencing experiments. Individual nanopore reads are shown in gray tick marks with reads >5 kb shown in dark red, and reads >2 kb shown in light red. Histograms of mean coverage of mapped bases across each bacterial genome from ticks sequenced during NAS enrichment is shown in orange, and coverage from ticks sequenced over control experiment shown in blue.

Supplementary Table 1. Whole genome and plasmid sequences of known and potential tick-borne pathogens included in enrichment FASTA file for adaptive sampling enrichment and real-time alignment.

Sequence Number	Organism	Group	Strain	Sequence Type	Accession Number	Length (bp)
1	*B. burgdorferi* s.s.	Bacteria	JD1	Complete Genome	CP002312.1	922,801
2	*B. burgdorferi* s.s.	Bacteria	JD1	Plasmid	CP002316.1	26,525
3	*B. burgdorferi* s.s.	Bacteria	JD1	Plasmid	CP002310.1	60,739
4	*B. burgdorferi* s.s.	Bacteria	JD1	Plasmid	CP002323.1	30,314
5	*B. burgdorferi* s.s.	Bacteria	JD1	Plasmid	CP002309.1	29,902
6	*B. burgdorferi* s.s.	Bacteria	JD1	Plasmid	CP002321.1	30,019
7	*B. burgdorferi* s.s.	Bacteria	JD1	Plasmid	CP002320.1	29,707
8	*B. burgdorferi* s.s.	Bacteria	JD1	Plasmid	CP002307.1	30,804
9	*B. burgdorferi* s.s.	Bacteria	JD1	Plasmid	CP002311.1	31,085
10	*B. burgdorferi* s.s.	Bacteria	JD1	Plasmid	CP002322.1	30,697
11	*B. burgdorferi* s.s.	Bacteria	JD1	Plasmid	CP002313.1	17,522
12	*B. burgdorferi* s.s.	Bacteria	JD1	Plasmid	CP002308.1	22,827
13	*B. burgdorferi* s.s.	Bacteria	JD1	Plasmid	CP002306.1	24,354
14	*B. burgdorferi* s.s.	Bacteria	JD1	Plasmid	CP002314.1	28,797
15	*B. burgdorferi* s.s.	Bacteria	JD1	Plasmid	CP002318.1	30,096
16	*B. burgdorferi* s.s.	Bacteria	JD1	Plasmid	CP002317.1	24,614
17	*B. burgdorferi* s.s.	Bacteria	JD1	Plasmid	CP002324.1	26,981
18	*B. burgdorferi* s.s.	Bacteria	JD1	Plasmid	CP002319.1	29,993
19	*B. burgdorferi* s.s.	Bacteria	JD1	Plasmid	CP002315.1	22,808
20	*B. burgdorferi* s.s.	Bacteria	JD1	Plasmid	CP002325.1	27,590
21	*B. burgdorferi* s.s.	Bacteria	JD1	Plasmid	CP001652.1	52,916
22	*A. phagocytophilum*	Bacteria	JM	Complete Genome	NC_021880.1	1,481,598
23	*Ba. microti*	Protozoa	RI	Chromosome 1	FO082871.1	1,304,281
24	*Ba. microti*	Protozoa	RI	Chromosome 2	FO082872.1	1,508,385
25	*Ba. microti*	Protozoa	RI	Chromosome 3	LN871598.1	1,766,409
26	*Ba. microti*	Protozoa	RI	Chromosome 4	LN871599.1	1,816,206
27	*Ba. microti*	Protozoa	RI	Mitochondrial Genome 1	LN871602.1	10,547
28	*Ba. microti*	Protozoa	RI	Mitochondrial Genome 2	LN871600.2	11,149
29	*Ba. microti*	Protozoa	RI	Mitochondrial Genome 3	LN871601.1	10,547
30	*Ba. microti*	Protozoa	RI	Mitochondrial Genome 4	LN871603.1	10,547
31	*Ba. microti*	Protozoa	RI	Apicoplast Genome	LK028575.1	28,657
32	*E. muris*	Bacteria	AS145	Complete Genome	CP006917.1	1,196,717
33	*B. miyamotoi*	Bacteria	LB-2001	Complete Genome	CP006647.2	907,293

34	*B. miyamotoi*	Bacteria	LB-2001	Plasmid	CP010328.2	30,673
35	*B. mayonii*	Bacteria	MN14-15339	Complete Genome	CP015796.1	904,387
36	*B. mayonii*	Bacteria	MN14-15339	Plasmid	CP015797.1	26,835
37	*B. mayonii*	Bacteria	MN14-15339	Plasmid	CP015798.1	30,433
38	*B. mayonii*	Bacteria	MN14-15339	Plasmid	CP015799.1	27,866
39	*B. mayonii*	Bacteria	MN14-15339	Plasmid	CP015800.1	30,330
40	*B. mayonii*	Bacteria	MN14-15339	Plasmid	CP015801.1	30,406
41	*B. mayonii*	Bacteria	MN14-15339	Plasmid	CP015802.1	17,017
42	*B. mayonii*	Bacteria	MN14-15339	Plasmid	CP015962.1	8,307
43	*B. mayonii*	Bacteria	MN14-15339	Plasmid	CP015803.1	23,759
44	*B. mayonii*	Bacteria	MN14-15339	Plasmid	CP015804.1	24,696
45	*B. mayonii*	Bacteria	MN14-15339	Plasmid	CP015805.1	19,566
46	*B. mayonii*	Bacteria	MN14-15339	Plasmid	CP015806.1	27,809
47	*B. mayonii*	Bacteria	MN14-15339	Plasmid	CP015807.1	32,269
48	*B. mayonii*	Bacteria	MN14-15339	Plasmid	CP015808.1	45,035
49	*B. mayonii*	Bacteria	MN14-15339	Plasmid	CP015809.1	53,343
50	Powassan virus	Virus	ctb30	Complete Genome	AF311056.1	10,800
51	*Dirofilaria immitis*	Helminth	N/A	Mitochondrial Genome	NC_005305.1	13,814
52	*Bartonella henselae*	Bacteria	MVT02	Complete Genome	LN879429.1	1,905,383
